# A mutually beneficial collaboration between the European Academy of Allergy and Clinical Immunology Junior Members and Clinical and Translational Allergy

**DOI:** 10.1186/s13601-016-0133-8

**Published:** 2016-12-01

**Authors:** Peter Valentin Tomazic, Anke Graessel, Diana Silva, Ibon Eguiluz-Gracia, George V. Guibas, Clive Grattan, Jean Bousquet, Olympia Tsilochristou

**Affiliations:** 1 Department of General Otorhinolaryngology, H&NS, Medical University of Graz, Graz, Austria; 2 ZAUM - Center of Allergy and Environment, Technische Universität and Helmholtz Center Munich, Munich, Germany; 3 Immunology Department, Faculty of Medicine, University of Porto, Porto, Portugal; 4 Serviço de Imunoalergologia, Centro Hospitalar de São João E.P.E., Porto, Portugal; 5 Allergy Department and Research Laboratory, Hospital Regional Universitario de Málaga and IBIMA, Málaga, Spain; 6 Division of Infection, Immunity and Respiratory Medicine, Allergy Department, University Hospitals South Manchester NHS Trust, The University of Manchester, Manchester, UK; 7 St John’s Institute of Dermatology, Guy’s Hospital, London, UK; 8 University Hospital, Montpellier, France; 9 MACVIA-France, Contre les MAladies Chroniques pour un VIeillissement Actif en France, European Innovation Partnership on Active and Healthy Ageing Reference Site, Montpellier, France; 10 INSERM, VIMA: Ageing and Chronic Diseases, Epidemiological and Public Health Approaches, U1168, Paris, France; 11 UMR-S 1168, Université Versailles St-Quentin-en-Yvelines (UVSQ), Paris, France; 12 Division of Asthma, Allergy and Lung Biology, Department of Paediatric Allergy, Children’s Allergy Service, King’s College London & Guy’s and St. Thomas’ National Health Service Foundation Trust, SE1 7EH London, UK

**Keywords:** EAACI, Junior Members, Clinical and Translational Allergy, Collaboration, Allergy

## Abstract

The European Academy of Allergy and Clinical Immunology (EAACI) Junior Members (JM) comprise the largest EAACI section with around 4000 clinicians and scientists under 35 years of age working in the field of allergy and clinical immunology. The Junior Member collaboration with Clinical and Translational Allergy Journal is a mutually beneficial relationship providing Junior Members of EAACI with excellent opportunities to publish their work in the Journal, enhance their visibility in their respective field, and get involved with Journal-related activities and processes. In the future, this collaboration will grow, not only by the consolidation of these activities, but also by the implementation of new initiatives, such as a platform for discussing and/or publishing Junior Members’ dissertations in the Journal. From the CTA perspective, the collaboration presents an opportunity to promote a new generation of allergists with experience of conducting and presenting research, with improved skills in critical review.

## Background

The European Academy of Allergy and Clinical Immunology (EAACI) Junior Members (JM) comprise the largest EAACI section with around 4000 clinicians and scientists under 35 years of age working in the field of allergy and clinical immunology. EAACI JM benefit from free EAACI membership, reduced registration fees for the EAACI events (Annual Congress, Focused Meetings and Allergy Schools), free online access to the EAACI Journals and educational resources and the possibility of applying for the EAACI Mentorship Programme, Clinical and Research Fellowships, Scholarships and travel grants. The JM working group (WG), founded in 2001 [1], is the representative body of the JM within EAACI as an organisation. The JM WG has been receiving support and encouragement from the EAACI leadership and has thus been able to coordinate a number of initiatives such as: (a) Involvement in the organization and planning of sessions for the EAACI Congresses, Focused Meetings and Allergy Schools. In particular, the JM WG operates its own programme at all EAACI Congresses, which apart from scientific meetings involves several social activities that help juniors get to know each other better in a more relaxed environment (b) Establishment of JM social media accounts (on Facebook, Twitter and LinkedIn) with the aim of increasing the flow of information, visibility, communication and networking among JM. (c) Development and update of the JM dedicated webpages on eaaci.org. (d) The Mentorship Programme supporting the communication between seniors and juniors. (e) Coordination and execution of translations of official EAACI publications from English into other main European languages e.g. “A new framework for the interpretation of IgE sensitization tests” [2], the International Consensus documents on Drug Allergy [3] and Pediatric Asthma [4] as well as resources and webpages on EAACI’s campaign (e.g. bewareofallergy.com); and (f) Collaboration with the Editorial Boards of the three EAACI Journals, namely the European Journal of Allergy and Clinical Immunology (Allergy), Pediatric Allergy and Immunology (PAI), Clinical and Translational Allergy (CTA), with the aim of promoting the articles published by these Journals.

The primary aim of Clinical and Translational Allergy is to publish high quality translational research in allergy for clinicians and their patients using an open access platform to allow unrestricted availability and dissemination through social media. A secondary objective is to promote high standards of critical assessment of manuscripts through the review process by EAACI Junior Members.

## The JM-CTA collaboration

Clinical and Translational Allergy, is the youngest journal in the portfolio of EAACI and provides a platform for the dissemination of allergy research and reviews, EAACI task force reports, position papers and guidelines amongst an international scientific audience. It accepts clinical and translational research articles in the field of allergy and immunology and all its articles are open-access and exclusively web-based, thus ensuring a wide dissemination of its content.

The JM-CTA collaboration aims at promoting the Journal’s scientific output and increasing its visibility amongst the EAACI Junior and Senior members as well as the general allergy-interested community. In addition, JM can extend their influence and input within the Journal. The juniors that comprise the JM-CTA collaboration team are members of the JM WG and are responsible for:

- Monthly selection of the CTA ‘JM Must Read articles‘; these articles are specifically flagged on the CTA homepage, because they are most relevant to fellows in training based on their educational content. Due to the open access policy of the Journal, all juniors can easily download these articles.
- Active promotion of CTA in the most popular social media networks; all recent and past, highly accessed articles are posted on EAACI’s Facebook and Twitter account with the hashtag #CTA_Journal.
- Organization and development of webcasted interviews; leading authors of recently published *CTA* articles are invited to be interviewed by a JM. The purpose of this initiative is to promote the article, give JMs the opportunity to meet senior authors personally and discuss details of their work with them. The first interview [5] is already available on the EAACI YouTube channel and social media platforms as well as on CTA’s homepage.

The CTA Editorial Board has embraced all these initiatives and has enhanced them further. Acknowledging the importance of education for the junior allergy physicians and researchers, the CTA Editorial Board supported the following actions:

- Highlighted the CTA ‘JM Must Read articles’ with the EAACI JM logo on CTA’s homepage.
- Implemented a Twitter live feed in relation to all tweets carrying the hashtag #CTA_Journal on the Journal’s website ensuring quick information access even to web-users not signed up for Twitter.
- A limited number of full waivers of the Article Processing charge (APC) will be available to EAACI JM’s to publish their original research in addition to Academy position papers and guidelines.
- Invited qualified EAACI JM to act as reviewers for the Journal. This not only underlines their recognition in the field but also enhances their critical view on research.
- Launched two quizzes that were created by the JM of the JM-CTA collaboration team in relation to articles published in CTA [6, 7]. Upon successful completion, the Journal offered waived publication fees for two articles submitted by the two JM quiz winners.
- Released an open call to submit hot topic reviews with Juniors as first authors. Four of these articles have been published in CTA within the last year. Saluja et al. [8] published about the role of interleukin-33 in allergy and inflammation. Ozyigit et al. [9] described the role of innate lymphoid cells in asthma. Also, reviews with a more clinical and translational research orientation were published through this initiative; Song and Chang [10] published a review dealing with cough hypersensitivity as a neuro-immune interaction, while Schrijvers et al. [11] focused on the pathogenesis and diagnosis of delayed-type drug hypersensitivity reactions.

## Conclusion

The Junior Member collaboration with Clinical and Translational Allergy Journal is a mutually beneficial relationship providing Junior Members of EAACI with excellent opportunities to publish their work in the Journal, enhance their visibility in their respective field, and get involved with Journal-related activities and processes. In the future, this collaboration will grow, not only by the consolidation of these activities, but also by the implementation of new initiatives, such as a platform for discussing and/or publishing Junior Members’ dissertations in the Journal. From the CTA perspective, the collaboration presents an opportunity to promote a new generation of allergists with experience of conducting and presenting research, with improved skills in critical review.

## Abbreviations

APC: Article Processing charge
CTA: Clinical and Translational Allergy Journal
EAACI: European Academy of Allergy and Clinical Immunology
JM: Junior Members
PAI: Pediatric Allergy and Immunology Journal
WG: working group
